# Impact of tumor necrosis factor α inhibitors on MRI inflammation in axial spondyloarthritis assessed by Spondyloarthritis Research Consortium Canada score: A meta-analysis

**DOI:** 10.1371/journal.pone.0244788

**Published:** 2020-12-31

**Authors:** Yupeng Huang, Yuehong Chen, Tao Liu, Sang Lin, Geng Yin, Qibing Xie

**Affiliations:** Department of Rheumatology and Immunology, West China Hospital, Sichuan University, Chengdu, Sichuan, China; Universiteit Antwerpen, BELGIUM

## Abstract

Spondyloarthritis Research Consortium Canada (SPARCC) score is an effective magnetic resonance imaging (MRI) evaluation method for inflammation in axial spondyloarthritis. Previously published meta-analyses have shown tumor necrosis factor α inhibitors (TNFi) had great effectiveness on improving disease activity and function in axial spondyloarthritis. However, there still has no one that concentrates on the impact of TNFi on MRI inflammation. We conduct a meta-analysis to summarize the impact of TNFi on MRI inflammation in axial spondyloarthritis using SPARCC score. Comprehensive search was conducted in the databases of OVID Medline, OVID EMBASE, and Cochrane library on November 14, 2020. We investigated the differences in SPARCC score of sacroiliac joint and spine, before and after TNFi treatment in patients with axial spondyloarthritis. SPARCC score was further compared in the subgroup by diagnostic category and TNFi types. In addition, clinical assessment indicators including ankylosing spondylitis disease activity score, bath ankylosing spondylitis disease activity index, bath ankylosing spondylitis functional index, c-reactive protein were also analyzed. Data were pooled by mean differences (MD) with 95% confidence intervals (CI) and publication bias was assessed by Egger’s test. Jadad scale was applied to assess the quality of included trials. Compared with control group, TNFi significantly improved SPARCC score of sacroiliac joints (n = 11, MD = 2.86, 95% CI 2.50, 3.23) and spine (n = 5, MD = 1.87,95%CI 1.27, 2.46). This effect was consistent among subgroups by different diagnostic category (ankylosing spondylitis, non-radiographic axial spondyloarthritis) and TNFi types (adalimumab, certolizumab pegol). Analysis of clinical assessment indicators also confirmed the therapeutic effect on axial spondyloarthritis. Egger’s test suggested no possibility of publication bias. This meta-analysis shows that TNFi are effective to improve MRI inflammation in patients with axial spondyloarthritis and the treatment effectiveness is not affected by diagnostic category and TNFi types.

## Introduction

Spondyloarthritis (SpA), a group of chronic inflammatory rheumatic diseases, affect joints, organs, and other tissues. Axial spondyloarthritis (axSpA) is the umbrella term for this group of diseases where the predominant involvement is in the axial skeleton. AxSpA can be further classified to radiographic axSpA, also termed ankylosing spondylitis (AS), and non-radiographic axial spondyloarthritis (nr-axSpA) based on whether definitive structural changes are visible on plain radiographs of the sacroiliac joints (SIJ). Both are characterized by inflammatory pain and functional impairment [1–4].

As the first-line medication for axSpA, non-steroidal anti-inflammatory drugs (NSAIDs) are well recognized for relieving acute symptoms and improving function [5, 6]. But the impact of NSAIDs on radiographic progression was very limited [5] and trial data have arose doubts of NSAIDs on preventing the potential structural progress [7–10]. Local injections of glucocorticoid to the site of musculoskeletal inflammation may be considered an option to treat arthritis and enthesitis [5]. A prior study suggested that short-term glucocorticoid had a mild effect on signs and symptoms [11], but long-term treatment of systemic glucocorticoids was not recommended in patients with axial disease [5]. In spite of the therapeutic effect on rheumatoid arthritis, conventional synthetic disease-modifying antirheumatic drugs (csDMARDs), such as sulfasalazine, methotrexate, and leflunomide have no proven efficacy for axSpA [12–15]. Biological disease-modifying antirheumatic drugs (bDMARDs) are the milestones for the treatment of axSpA [5, 6, 16] Tumor necrosis factor α inhibitors (TNFi) including etanercept, adalimumab, certolizumab pegol, infliximab, and golimumab have shown effect in improving disease activity and physical function [17–19].

To detect and supervise inflammation or structure damage of the joints, among the several available radiology methods, such as X-ray, computed tomography (CT), magnetic resonance imaging (MRI), MRI is considered as the best imaging technique [20], which shows a high sensitivity on detecting the inflammation lesion generally manifested as bone marrow edema (BME) at SIJ and spine [21, 22]. In recent years, MRI has become an important evaluation method for axSpA, especially in patients with inconclusive findings or negative findings on X-rays. The AS working group of the International Association for the Evaluation of Spinal Arthritis (ASAS)/Outcome Measures in Rheumatology (OMERACT) had proposed that MRI should be applied as the first choice to evaluate spinal arthritis [23]. The Spondyloarthritis Research Consortium Canada (SPARCC) score suggested by Landewé et al. [24] had a high sensitivity to reflect inflammation changes and displayed a positive correlation to clinical disease activity. The scoring system was developed by Maksymowych for assessing the inflammatory activity in SIJ and spine relied on the short time inversion recovery (STIR) sequence [25, 26]. For evaluation of inflammation in SIJ reflected by BME, the SPARCC score evaluate the SIJ with highest inflammatory activity, dividing the bilateral joint into 4 quadrants: upper iliac, lower iliac, upper sacral, and lower sacral. Each of these 4 quadrants was scored on a dichotomous way, where 1 = presence of BME and 0 = absent edema signal. Additional score of 1 was given when both sides of joint with a BME signal of depth≥1 cm from the articular surface or a intense signal respectively. The maximal score for a single coronal slice is 12 and the scoring system was repeated in 6 consecutive coronal slices, leading to a total score of 72 [25]. The SPARCC spine score assesses the BME in vertebral bodies of spine based on 6 most severely affected discovertebral unit (DVUs) in 3 consecutive coronal slices and its grading method is similar with the SIJ score, bringing maximum score to 108 [26].

Previously published meta-analysis mainly concerned on the treatment effect of TNFi on disease activity and function in axSpA patients assessed by clinical evaluation methods such as Bath Ankylosing Spondylitis disease activity index (BASDAI), Bath Ankylosing Spondylitis functional index (BASFI) [27, 28], while no one concentrated on the MRI inflammation. To summarize the impact of TNFi on MRI inflammation assessed by SPARCC score, we performed the meta-analysis, accompanied with other assessment indicators including Ankylosing Spondylitis Disease Activity Score (ASDAS), BASDAI, BASFI, and C-reactive protein (CRP).

## Materials and methods

### Search strategy

Literature retrieval was performed in electronic databases of OVID Medline, OVID EMBASE, and Cochrane library on November 14, 2020. The combination search of key words and MeSH terms were conducted and the search terms were ‘spondyloarthritis’, ‘tumor necrosis factor’ and ‘magnetic resonance imaging’. In addition, references of included studies were also manually checked to identify possibly eligible studies. Detailed search strategy was presented in S1 File. Two authors respectively screened the titles and abstracts, and read the full texts according to predefined study selection criteria. Disagreement was resolved by consensus.

### Study selection

The eligible studies met all the following criteria: (1)Study patients fulfilled the spondyloarthritis international society (ASAS) classification criteria for axSpA [29] or the modified New York criteria [30]; (2) Patients in treatment group received one of the five TNFi (etanercept, adalimumab, certolizumab pegol, infliximab, and golimumab) while in control group patients were treated with placebo, NSAIDs, csDMARDs, or any non-TNFi biologics; (3) Using SPARCC score to assess the treatment effect at sites of spine (scale 0–108) and SIJ (scale 0–72), accompanied with other therapeutic effect indicators including ASDAS, BASDAI, BASFI, CRP. (4) Study design was a randomized controlled trial.

A study would be excluded if it met any one of the following statements: (1) a duplicate study; (2) no data related to SPARCC score; (3) TNFi were used in both groups; (4) a letter or a conference abstract without full text.

### Data extraction

Two authors independently extracted data and disagreements were addressed by discussion and re-evaluation. We collected information on family name of first author, year of publication, country of first author, subgroup diagnosis of axSpA, treatment information, study duration, and study information in both treatment group and control group (e.g., patient number, mean age, disease duration, interventions, baseline and endpoint or changes of therapeutic effect indicators).

### Quality assessment

Jadad scale was applied to assess the methodology quality of included studies [31], containing randomization, double blinding, and drop out and loss of follow-up. For randomization, scores of 0, 1, 2 are given for no mention or inappropriate randomization methods (e.g., based on even or odd number of birth date or admission order), inadequate description like randomization without specific methods, adequate and appropriate randomization (e.g., randomization by random number table or computer). For double blinding, 0 is given to no blinding or inappropriate double binding method, 1 for inadequate description of double blinding, and 2 for adequate and appropriate description of double binding. For drop out and loss of follow-up, 0 is given to no description while 1 is for adequate description. For each study, the highest score is 5, and score less than 2 is considered as low study quality while higher than 3 is deemed as high study quality.

### Statistical analysis

Data were analyzed by Review Manager 5.3. The major outcomes were SPARCC score at SIJ and spine. Other outcomes including ASDAS, BASDAI, BASFI, and CRP were also studied when data were available. Data were pooled by the mean difference (MD) with 95% confidence interval (CI). Heterogeneity among the studies was evaluated by I^2^ statistic and P value at 0.1. Fixed-effect model was adopted when there was no or low heterogeneity (I^2^≤50%) while random-effect model was applied when there was high heterogeneity (I^2^>50%).

Subgroup meta-analyses by diagnostic category (AS and nr-axSpA) and TNFi types (etanercept, adalimumab, golimumab, certolizumab pegol, infliximab and golimumab) were also performed (if data were available). Sensitivity analysis was conducted to test the robustness of the outcomes. Egger’s test was applied to assess the publication bias.

## Results

### Study selection result

**Fig 1:**
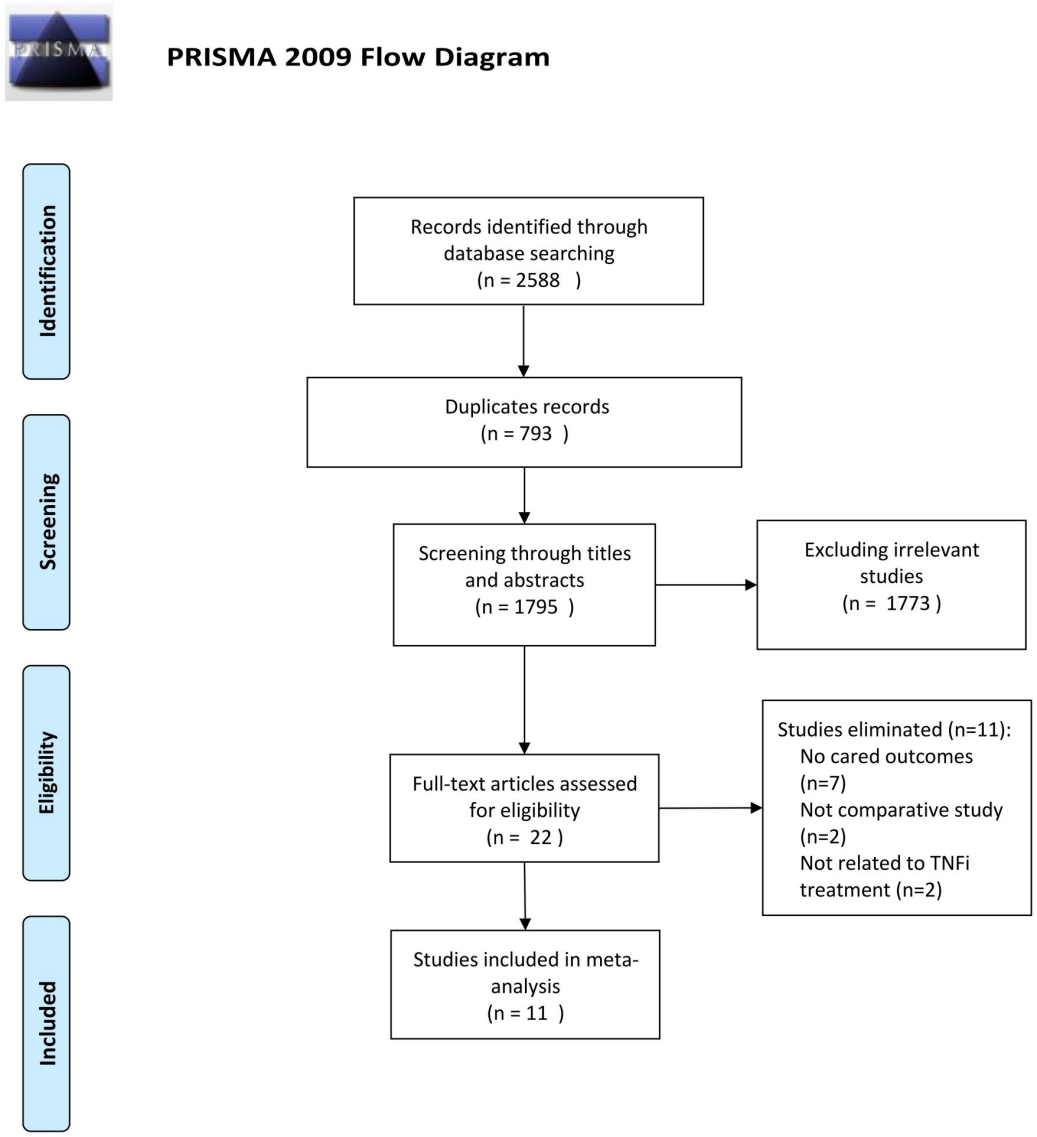
Study selection process. *From*: Moher D, Liberati A, Tetzlaff J, Altman DG, The PRISMA Group (2009). *P*referred *R*eporting *I*tems for *S*ystematic Reviews and *M*eta-*A*nalyses: The PRISMA Statement. PLoS Med 6(7): e1000097. doi:10.137l/journal.prned1000097. **For more information, visit** www.prisma-statement.org.

A total of 2588 studies were retrieved from the electronic databases of OVID Medline (n = 410), OVID Embase (n = 1911) and Cochrane library (n = 267). After excluding duplicate studies (n = 793), eliminating irrelevant studies by screening titles and abstracts (n = 1773), and deleting unmet selection criteria studies by reading full texts, 11 studies [32–42] with a total of 685 patients in TNFi group and 638 patients in control group were included (Fig 1). Manually checking the references of included studies did not find extra eligible studies.

### Study characteristics of included studies

The study characteristics of included studies were summarized in Table 1. Of the 11 included studies, three studies were performed in Germany, two in China, two in Denmark, one in Canada, one in Hong Kong, one in United States and one in France; two studies reported data in AS patients, five studies focused on patients with axSpA, four on nr-axSpA patients; in the treatment group, five trials focused on adalimumab, two on golimumab, two on certolizumab pegol, one on etanercept, and one did not specify individual TNFi; in the control group, nine controls were placebo, one was pamidronate, and one was csDMARDs. Among the included studies, 10 studies clearly pointed out that patients were treated unsuccessfully with ≥ 1 NSAIDs before participation. Other type of biological therapy was not permitted before enrollment. Concomitant treatments, including NSAIDs, csDMARDs, and prednisone, were reported in ten studies. Follow-up duration ranged from 12 weeks to 104 weeks, median 48 weeks. In total, eleven studies reported data on SPARCC score at SIJ concerning the comparison between TNFi group and control group and five studies reported data of SPARCC score at spine. The Jadad scales of all included studies were higher than 3.

**Table 1:** Characteristics of included studies.

Included studies	Country	Diagnosis	Concomitant treatment	Follow up duration (weeks)	TNFi group					Control group					Jadad score
					Patient number (male %)	Age (mean ± SD, years)	Disease duration (mean ± SD, years)	TNFi (Usage)	Baseline SPARCC (sacroiliac joint/spine)	Patient number (male %)	Age (mean ± SD, years)	Disease duration (mean ± SD, years)	Controls, (Usage)	Baseline SPARCC (sacroiliac joint/spine)	
**Lambert 2007** [32]	Canada	AS	SSZ, HCQ, MTX, Pred, NSAIDs	52	38 (81.8%)	40±10.9	12.1±8.7	Adalimumab (40 mg every other week)	5.7±9.0/16.0±15.6	44 (76.3%)	41.9±11.1	14.5±9.0	Placebo	7.5±10.0/19.9±19.8	5
**Hu 2012** [35]	China	AS	SSZ, MTX, Pred, NSAIDs	24	26 (92.3%)	28.2±6.9	7.4±5.7	Adalimumab (40 mg every other week)	10.1±9.5/17.0±12.2	20 (100%)	27.4±7.2	7.6±4.6	Placebo	9.0±9.1/19.7±12.7	5
**Sieper 2013** [33]	Germany	nr-axSpA	AZA, SSZ, HCQ, MTX, Pred, NSAIDs	12	91 (48%)	37.6±11.3	10.1±9.0	Adalimumab (40 mg every other week)	5.1±6.2/4.1±5.5	94 (43%)	38.4±10.4	10.1±8.8	Placebo	4.7±5.9/4.6±6.0	5
**Sieper 2015** [34]	Germany	nr-axSpA	NSAIDs	16	98 (62.2%)	30.7±7.1	NR	Golimumab (50mg every 4 weeks)	9.9±11.82/NR	100 (52%)	31.7±7.2	NR	Placebo	12.7±15.62/NR	5
**Mok 2015** [38]	Hong Kong	axSpA	NSAIDs	48	20 (80%)	32±10.7	4.2±3.3	Golimumab (50mg every 4 weeks)	15.8±17.7/11.4±10.8	10 (90%)	36.3±11.4	5.0±3.7	Pamidronate (60mg every 4 weeks)	8.25±6.16/12.4±13.6	5
**Cui 2016** [41]	China	axSpA	NSAIDs	52	18 (16.6%)	23±NR	0.67±NR	TNFi(NR)	27.76±18.38/NR	17 (17.6%)	28±NR	7.5±NR	DMARDs (NR)	28.67±15.51/NR	3
**Pedersen 2016** [36]	Denmark	axSpA	NSAIDs	48	25 (77.8%)	39.6±12.4	10.9±10.8	Adalimumab (40 mg every other week)	6.3±9.1/NR	27 (76%)	37.5±9.4	8.2±8.1	Placebo	10.6±12.2/NR	5
**Braun 2017** [40]	Germany	axSpA	NSAIDs, DMARDs	96	109 (62.3%)	38.9±NR	NR	Certolizumab pegol (400 mg at weeks 0, 2 and 4 followed by either CZP 200 mg every 2 weeks or CZP 400 mg every 4 weeks)	6.9±10.4/NR	54 (68.5%)	38.9±NR	NR	Placebo	12.9±16.6/NR	4
**Dougados 2017** [39]	France	nr-axSpA	NR	104	106 (62%)	31.9±7.8	2.4±1.9	Etanercept (50mg once weekly)	8.0±9.7/4.7±7.1	109 (53%)	32.0±7.8	2.5±1.8	Placebo	7.7±10.1/3.5±5.6	5
**Hededal 2017** [37]	Denmark	axSpA	NSAIDs	12	27 (75%)	40.8±12.6	11.5±7.2	Adalimumab (40 mg every other week)	4.8±10.4/NR	27 (77.8%)	38.3±9.5	10.2±9.1	Placebo	10.1±14.7/NR	4
**Deohar 2019** [42]	United States	nr-axSpA	NSAIDs, DMARDs, corticosteroid	52	159(49.1%)	37.3±10.5	7.8±7.7	Certolizumab pegol (400 mg at weeks 0, 2, and 4,followed by 200 mg every 2 weeks)	7.79±10.82/NR	158(48.1%)	37.4±10.8	8.0±7.5	Placebo	8.46±12.31/NR	5

Abbreviations: AS, ankylosing spondylitis; axSpA, axial spondyloarthritis; nr-axSpA, non-radiographic axial spondyloarthritis; NSAIDs, non-steroidal anti-inflammatory drugs; DMARDs, disease-modifying antirheumatic drugs; Pred, prednisone; AZA, azathioprine; SSZ, sulfasalazine; HCQ, hydroxychloroquine; MTX, methotrexate; NR, not report; SD, standard deviation; TNFi,tumor necrosis factor α inhibitor; SPARCC, Spondyloarthritis Research Consortium Canada score.

### Treatment outcomes

**Fig 2:**
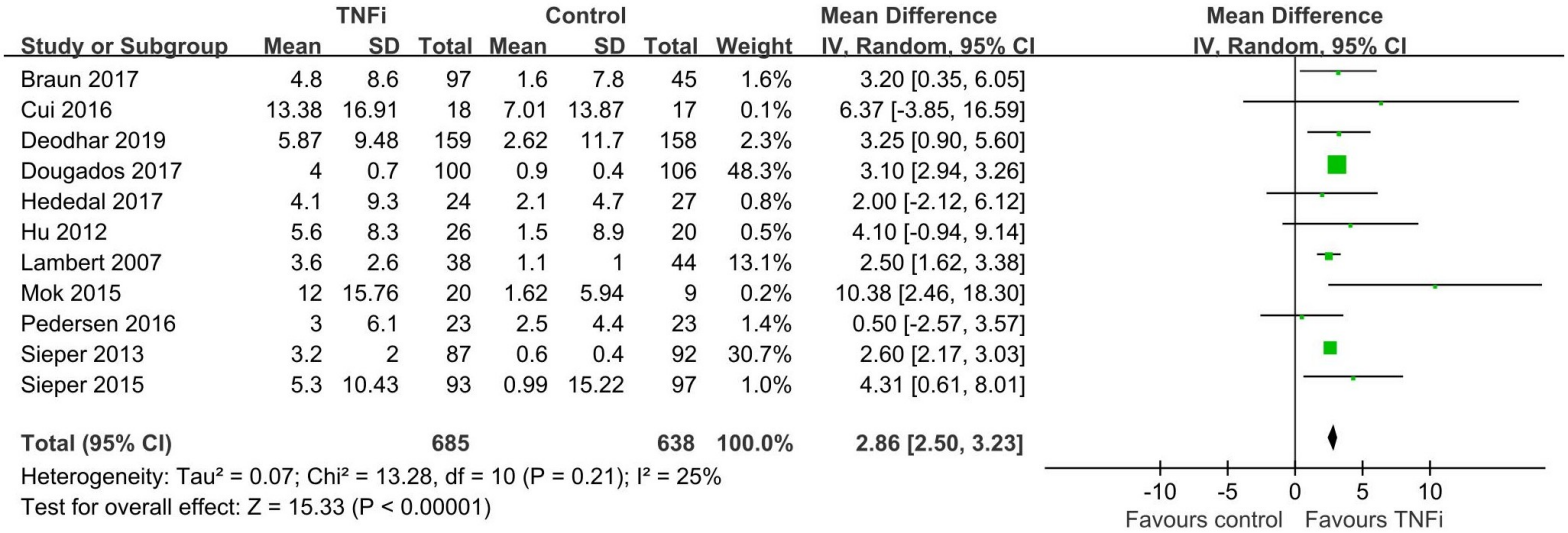
Forest plot of SPARCC SIJ score based on TNFi treatment versus controls.

**Fig 3:**
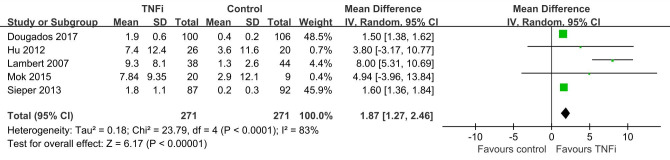
Funnel plot of SPARCC spine score on the basis of TNFi versus controls.

In total, eleven studies [32–42] enrolled 1323 patients investigated SPARCC score of SIJ which reported TNFi were better to reduce MRI inflammation than controls (n = 11, MD = 2.86, 95% CI 2.50, 3.23) (Fig 2). Five studies [32, 33, 35, 38, 39] involving 542 patients suggested that SPARCC spine score achieved a better reduction in TNFi group (n = 5, MD = 1.87,95%CI 1.27, 2.46) (Fig 3). Similar to the SPARCC score of SIJ and spine, treatment effects of TNFi assessed by ASDAS, BASDAI, BASFI and CRP were more effective than controls (n = 7, MD = 0.96, 95% CI 0.71, 1.20; n = 7, MD = 1.05, 95% CI 0.61, 1.49; n = 6, MD = 0.88, 95% CI 0.56, 1.19; n = 7, MD = 3.87, 95% CI 1.09, 6.65) (Table 2).

**Table 2:** Pooled data of clinical indicators.

Outcomes (TNFi vs. Control)	Study number	Total patient number		Heterogeneity		MD (95%CI)	P value
		TNFi	Control	I^2^	P		
**ASDAS**	7	367	364	82%	P<0.00001	0.96[0.71, 1.20]	P<0.00001*
**BASDAI**	7	508	505	77%	0.0002	1.05[0.61, 1.49]	P<0.00001*
**BASFI**	6	485	482	52%	0.06	0.88[0.56, 1.19]	P<0.00001*
**CRP**	7	503	499	66%	0.007	3.87[1.09, 6.65]	P = 0.006*

Abbreviations: TNFi, tumor necrosis factor α inhibitor; ASDAS, Ankylosing Spondylitis Disease Activity Score; BASDAI, Bath Ankylosing Spondylitis disease activity index; BASFI, Bath Ankylosing Spondylitis functional index; CRP, C-reactive protein; MD, mean difference; CI, confidence interval;

*p<0.05.

### Subgroup analysis by diagnostic category

Subgroup analysis by diagnostic category was performed to test whether the impact of TNFi on MRI inflammation was consistent in nr-axSpA and AS. Data reported by more than two studies were pooled (Table 3). As compared to controls, TNFi were effectively to improve MRI inflammation regardless of disease subgroup assessed by SPARCC SIJ score (nr-axSpA: n = 4, MD = 2.94, 95% CI 2.56, 3.31; AS: n = 2, MD = 2.55, 95% CI 1.68, 3.41), SPARCC spine score (nr-axSpA: n = 2, MD = 1.52, 95% CI 1.41,1.63; AS: n = 2, MD = 7.18, 95% CI 3.92, 10.44). In terms of clinical indicators, TNFi were more effective in nr-axSpA to ameliorate ASDAS (n = 3, MD = 0.87, 95% CI 0.58, 1.16), BASDAI (n = 4, MD = 0.85, 95% CI 0.37, 1.33), BASFI (n = 4, MD = 0.90, 95% CI 0.51, 1.28) and CRP (n = 4, MD = 2.94, 95% CI 0.21, 5.67) in comparisons with controls.

**Table 3:** Pooled data of subgroup analysis by diagnostic category.

Outcomes (TNFi vs. Control)	Study number	Total patient number		Heterogeneity		MD (95%CI)	P value
		TNFi	Control	I^2^	P		
**SPARCC SIJ score**							
nr-axSpA	4	439	453	41%	0.16	2.94[2.56, 3.31]	P<0.00001*
AS	2	64	64	0%	0.54	2.55[1.68, 3.41]	P<0.00001*
**SPARCC spine score**							
nr-axSpA	2	187	198	0%	0.47	1.52[1.41,1.63]	P<0.00001*
AS	2	64	64	18%	0.27	7.18[3.92,10.44]	P<0.0001*
**ASDAS**							
nr-axSpA	3	280	295	91%	P<0.0001	0.87[0.58,1.16]	P<0.00001*
**BASDAI**							
nr-axSpA	4	439	453	84%	0.0003	0.85[0.37,1.33]	P = 0.0005*
**BASFI**							
nr-axSpA	4	439	453	71%	0.01	0.90[0.51, 1.28]	P<0.00001*
**CRP**							
nr-axSpA	4	439	453	73%	0.01	2.94[0.21,5.67]	P = 0.03*

Abbreviations: SPARCC, Spondyloarthritis Research Consortium Canada score; SIJ, sacroiliac joints; TNFi, tumor necrosis factor α inhibitor; ASDAS, Ankylosing Spondylitis Disease Activity Score; BASDAI, Bath Ankylosing Spondylitis disease activity index; BASFI, Bath Ankylosing Spondylitis functional index; CRP, C-reactive protein; AS, ankylosing spondylitis; axSpA, axial spondyloarthritis; nr-axSpA, non-radiographic axial spondyloarthritis; MD, mean difference; CI, confidence interval;

*p<0.05.

### Subgroup analysis by TNFi types

Subgroup analysis by TNFi types was conducted to assess whether different TNFi affected the treatment outcomes (Table 4). As compared to placebo, adalimumab (ADA) was more potently to reduce SPARCC SIJ score (n = 5, MD = 2.55, 95% CI 2.17, 2.93), ASDAS (n = 3, MD = 1.08, 95% CI 0.63, 1.53), BASFI (n = 2, MD = 0.58, 95% CI 0.09, 1.07), and CRP (n = 2, MD = 3.98, 95% CI 1.04, 6.91), but there were no differences in SPARCC spine score (n = 3, MD = 4.44, 95% CI -0.63, 9.51) and BASDAI (n = 3, MD = 0.85, 95% CI -0.32, 2.02). Similarly, Certolizumab pegol (CZP) was more potently to reduce SPARCC SIJ score (n = 2, MD = 3.23, 95% CI 1.42, 5.04). As compared to control, golimumab (GLM) was more effective to reduce BASFI (n = 2, MD = 1.75, 95% CI 0.93, 2.56) and CRP (n = 2, MD = 8.86, 95% CI 3.19, 14.54).

**Table 4:** Pooled data of subgroup analysis by TNFi types.

Outcomes (ADA/GLM/CZP vs. Control)	Study number	Total patient number		Heterogeneity		MD (95%CI)	P value
		TNFi	Control	I^2^	P		
**SPARCC SIJ score**							
ADA vs. Placebo	5	198	206	0%	0.7	2.55[2.17, 2.93]	P<0.00001*
CZP vs. Placebo	2	256	203	0%	0.98	3.23[1.42,5.04]	P = 0.0005*
**SPARCC spine score**							
ADA vs. Placebo	3	151	156	91%	P<0.0001	4.44[-0.63, 9.51]	P = 0.09
**ASDAS**							
ADA vs. Placebo	3	136	135	54%	0.11	1.08[0.63,1.53]	P<0.00001*
**BASDAI**							
ADA vs. Placebo	3	136	135	74%	0.02	0.85[-0.32,2.02]	P = 0.16
**BASFI**							
ADA vs. Placebo	2	113	112	0%	0.53	0.58[0.09,1.07]	P = 0.02*
GLM vs. Control	2	113	106	11%	0.29	1.75[0.93, 2.56]	P<0.0001*
**CRP**							
ADA vs. Placebo	2	113	112	0%	0.95	3.98[1.04,6.91]	P = 0.008**
GLM vs. Control	2	113	106	0%	0.87	8.86[3.19,14.54]	P = 0.002*

Abbreviations: SPARCC, Spondyloarthritis Research Consortium Canada score; SIJ, sacroiliac joints; TNFi, tumor necrosis factor α inhibitor; ASDAS, Ankylosing Spondylitis Disease Activity Score; BASDAI, Bath Ankylosing Spondylitis disease activity index; BASFI, Bath Ankylosing Spondylitis functional index; CRP, C-reactive protein; AS, ankylosing spondylitis; axSpA, axial spondyloarthritis; nr-axSpA, non-radiographic axial spondyloarthritis; ADA, Adalimumab; GLM, Golimumab; Certolizumab pegol (CZP); MD, mean difference; CI, confidence interval;

*p<0.05.

### Sensitivity analysis

Sensitivity analysis of treatment outcomes was performed by deleting those studies that controls were not placebo, resulting in comparing TNFi versus placebo and results showed the treatment effect of TNFi versus placebo were consistent with TNFi versus controls, suggesting the results of the study were robust (S1 Table).

### Publication bias

We assessed the public bias using Egger’s test, suggesting there had no possibility for potential publication bias in the analysis of SPARCC SIJ score(P = 0.320) and spine score(P = 0.249).

## Discussion

This meta-analysis is the first study to evaluate the impact of TNFi on MRI inflammation in axSpA patients and shows TNFi are capable of relieving MRI inflammation in SIJ and spine, as well as ASDAS, BASDAI, BASFI, and CRP. Inflammatory changes are objective signs of axSpA. Inflammation in the SIJ (sacroiliitis) and in spine (spondylitis) can be visualized as BME [20]. If the inflammation is not controlled, structural changes such as fat metaplasia, erosions, sclerosis, or ankylosis in SIJ and vertebral edge would appear in successive years, which is so called radiographic progression. Radiographic progression takes place in 20–45% of AS patients and 7% of nr-axSpA within 2 years [43, 44]. The role of TNFi in slowing radiographic progression is still unclear. A meta-analysis reported no significant effect of TNFi on delaying spinal radiographic progression in AS [45]. More recently, another meta-analysis showed that TNFi might slow radiographic progression at spine in AS patients with treatment time for more than 4 years but not for shorter time like 2 years [46]. Nevertheless, both the studies were concentrated on the change of modified Stoke AS Spine Score (mSASSS) for axial damage detected by conventional radiography.

Controlling symptoms and inflammation, preventing progressive structural damage, preserving joint function are the treatment goals of axSpA [5] and early detection of inflammatory is the key link of diagnosis and treatment. MRI, not only directly shows the edema signal of inflammation site, but also displays the bone erosion and osteosclerosis of joint surface, can sensitively find the edema of the bone marrow and the surrounding soft tissue of SIJ and spine [20, 47], and is capable of making a diagnosis of inflammation before the obvious morphological change in X-ray and CT [48]. Moreover, previous studies indicated that lesions identified by MRI had a predictive capacity for the development of sacroiliitis [49, 50]. In addition, MRI inflammation score could be a predictor for disease remission. A study by Sieper et al. revealed that higher SPARCC SIJ score and lower SPARCC spine score at baseline would predict ASDAS inactive disease at week 12 in nr-axSpA [51].

Application of TNFi in axSpA is recommended in the patients who failed to reach disease relief after NSAIDs treatment or had high CRP level [5, 6]. In our study, adalimumab is the most used drugs in the reviewed researches, followed by golimumab and Certolizumab pegol in axSpA patients. It is reported male are more common than female in patients with established AS while female are dominant in nr-axSpA and patients with established AS are tended to have increased CRP level, MRI inflammation, and more structure changes than patients with nr-axSpA [52]. Our results suggest TNFi are effective to treat axSpA regardless of the disease subgroups assessed by SPARCC score. Regrettably, treatment effect of TNFi in disease subgroup could not relate to gender owing to the limited data report. The novel biologic agents targeting interleukin (IL)-17 such as secukinumab and ixekizumab were superior to placebo with respect to improving objective inflammation assessed by MRI [53] and long-term IL-17 inhibitor exposure might slow down radiographic progression in axSpA patients [54]. Another treatment option is tofacitinib, an oral Janus kinase (JAK) inhibitor, achieving meaningful reductions in SIJ and spinal MRI inflammation in AS patients [55, 56]. But the effectiveness of tofacitinib on delaying radiographic progression is limited.

Minimally important change (MIC) of imaging score can be helpful to reflect treatment responses and understand the number of patients showing significant changes. Maksymowych et al. defined the MIC of SPARCC score as ≥2.5-point change for SIJ score, and≥5-point change for spine score [57]. The MIC of spine score indicates a change of 5 quadrants in DVUs with BME. Similarly, the MIC of SIJ score indicates a change of at least 2–3 quadrants with BME in sacroiliac joint. Among the enrolled 10 studies, changes of SPARCC SIJ score in TNFi group all exceeded the MIC, and three of five studies reported that the changes of SPARCC spine score were greater than MIC. In trials of nr-axSpA, the most serious inflammation located in the SIJ but almost no inflammation in the spine. Conversely, in trials of AS, inflammation mostly located in the spine [3]. As shown in our study, after TNFi treatment, the improvement of SPARCC SIJ score in nr-axSpA was greater than in AS. In contrast, the changes of SPARCC spine score had a better reduction in AS patients.

Several inflammation scoring methods based on MRI including Berlin, Aarhus, Leeds, and SPARCC have been reported. The Berlin method grades inflammation lesions according to the percent involvement of the bone marrow: 0: no high signal on quadrant area, 1: <33% of quadrant area, 2: high signal is ≥33% and <66% of the quadrant area, and 3: ≥66% of the quadrant area [58]. Similarly, both Aarhus and Leeds are based on semi-qualitative grading methods [59, 60]. SPARCC score is a quantitative method on sacroiliitis and spondylitis with excellent intraobserver and good interobserver reproducibility [25, 36]. Besides, SPARCC scoring system is reliable for measurement of enthesitis [61]. Afterwards the same research team developed and validated the SPARCC structure score for the assessment of structural lesions on MRI in the SIJ of patients with SpA [62], also with feasibility and reliability for pediatric SIJ MRI evaluation [63]. Newer measures, such as quantitative low-dose CT may provide higher sensitivity to find small changes in syndesmophyte size [64], and has a good correlation with Schober test in AS [65].

Five of 11 included studies had open-label period and data from open-label phase were not been analyzed in our study. It is often unclear what type of data is used for these trials (e.g. observed case or imputed data). Moreover, there is often considerable missing data at the point when the follow up MRI is typically performed. In addition, the control group will have received active treatment with TNFi in the open label period, the absolute changes in inflammation became evident in control group. So, this should be confused to compare the treatment effect in both groups in open-label phase. Thus, it is sufficient to show the data from analyses of the double-blind phase.

Heterogeneity was found among the studies in several indicators, which might originate from the following aspects. It is unclear whether the SPARCC method was correctly used in the different studies. As reported by the studies, Hu et al. [35] only calculated the SPARCC spine score from the lower T12 to upper S1 level instead of standardized method in which scoring the most serious involved 6 DVUs in the entire spine [26]. Additionally, we only searched studies in English, and some relevant studies in non-English might not be included. Small numbers of studies included in our analysis would limit the statistic power. Furthermore, the included studies had different inclusion and exclusion criteria, causing the heterogeneity in the efficacy of TNFi between studies. At last, our study was unable to distinguish whether the dose in TNFi group were responsible for treatment effects.

## Conclusion

TNFi are effective to improve MRI inflammation of SIJ and spine in axSpA patients and treatment effectiveness is consistent among different diagnostic category and TNFi types. SPARCC score is a reliable way to reflect the treatment impact of TNFi on MRI-detected inflammation. Nevertheless, more studies are needed to warrant the results owing to small study number.

## Supporting information

S1 Checklist PRISMA 2009 checklist. (DOC)

S1 File Search strategy. (DOCX)

S1 Table Sensitivity analysis. (DOCX)
