# Supplementary material for: Impact of tumor necrosis factor α inhibitors on MRI inflammation in axial spondyloarthritis assessed by Spondyloarthritis Research Consortium Canada score: A meta-analysis
Source: PLoS One. 2020 Dec 31;15(12):e0244788. doi: 10.1371/journal.pone.0244788 (PMC7775088; doi:10.1371/journal.pone.0244788)
Supplement: S1 File — (DOCX) [file pone.0244788.s002.docx]

Searching date: November 14, 2020

**OVID Medline** (1946 to November Week 1 2020, n=410)：

1 ankylosing spondylitis.mp. or exp Spondylitis, Ankylosing/ (17640)

2 exp SPONDYLITIS/ or spondylitis.mp. (37305)

3 Spondylarthropathies.mp. or exp SPONDYLARTHROPATHIES/ or exp Arthritis,

Psoriatic/ (24514)

4 (ankylos$ or spondyl$).tw. (39123)

5 (bekhterev$ or bechterew$).tw. (735)

6 (Marie adj struempell$).tw. (7)

7 1 or 2 or 3 or 4 or 5 or 6 (56042)

8 "tumor necrosis factor-alpha".mp. or exp Tumor Necrosis Factor-alpha/ (155094)

9 ("tumor necrosis factor-alpha" or "tumour necrosis factor-alpha").mp. (159991)

10 tumor necrosis factor alpha antibody.mp. (152)

11 ("tumor necrosis factor-α" or "tumour necrosis factor-α").mp. (192797)

12 ("tumo?r necrosis factor-alpha" or "tumo?r necrosis factor-α").ti,ab. (130172)

13 ("tumor necrosis factor alpha antibody" or "tumor necrosis factor-α

antibody").mp. (218)

14 ("tumo?r necrosis factor alpha antibody" or "tumo?r necrosis factor-α

antibody").ti,ab. (258)

15 ("tumo?r necrosis factor* inhibitor*" or "tumo?r necrosis factor* antibod*").ti,ab.

(1175)

16 ("tumour necrosis factor alpha antibody" or "tumour necrosis factor-α

antibody").mp. (42)

17 ("tumor necrosis factor*" or "tumour necrosis factor*").mp. (193865)

18 ("tumor necrosis factor antibody" or "tumour necrosis factor antibody").mp.

(81)

19 "tumo?r necrosis factor?".ti,ab. (130514)

20 ("tumo?r necrosis factor alpha antibody" or "tumo?r necrosis factor-α

antibody").ti,ab. (258)

21 ("tumo?r necrosis factor* inhibitor*" or "tumo?r necrosis factor* antibod*").ti,ab.

(1175)

22 "anti-tumo?r necrosis factor*".ti,ab. (4553)

23 ("tumor necrosis factor antibody" or "tumour necrosis factor antibody").mp. (81)

24 ("tumor necrosis factor* inhibitor*" or "tumour necrosis factor* inhibitor*").mp.

(1643)

25 "anti‐TNF‐alpha monoclonal antibod*".ti,ab. (0)

26 anti tnf*.ti,ab. (10595)

27 anti-tnf*.ti,ab. (10595)

28 antitnf*.ti,ab. (38)

29 etanercept.mp. or exp ETANERCEPT/ (7750)

30 (TNFR:Fc or etanercept or p75-Fc or Enbrel).ti,ab. (6157)

31 certolizumab pegol.mp. or exp Certolizumab Pegol/ (850)

32 (certolizumab or CDP870 or Cimzia).ti,ab. (831)

33 golimumab.mp. (1108)

34 (golimumab or CNTO 148 or simponi).ti,ab. (830)

35 infliximab.mp. or exp INFLIXIMAB/ (13242)

36 (infliximab or cA2 or TA-650 or remicade).ti,ab. (147771)

37 adalimumab.mp. or exp ADALIMUMAB/ (7220)

38 (adalimumab or humira or d2e7).ti,ab. (5594)

39 8 or 9 or 10 or 11 or 12 or 13 or 14 or 15 or 16 or 17 or 18 or 19 or 20 or 21 or

22 or 23 or 24 or 25 or 26 or 27 or 28 or 29 or 30 or 31 or 32 or 33 or 34 or 35 or 36

or 37 or 38 (340023)

40 exp Magnetic Resonance Imaging/ or nuclear magnetic resonance imaging.mp.

(460111)

41 (MR imag* or magnetic resonance imag* or MRI).ti,ab. (350997)

42 40 or 41 (542667)

43 7 and 39 and 42 (411)

44 limit 43 to yr="2000 -Current" (410)

**OVID EMbase** (1974 to 2020 November 13, n=1911):

1 ankylosing spondylitis.mp. or exp ankylosing spondylitis/ (32852)

2 spondylitis.mp. or exp spondylitis/ (46544)

3 Spondylarthropathies.mp. or exp spondyloarthropathy/ (28324)

4 (ankylos$ or spondyl$).tw. (62536)

5 (bekhterev$ or bechterew$).tw. (678)

6 (Marie adj struempell$).tw. (0)

7 1 or 2 or 3 or 4 or 5 or 6 (92435)

8 "tumor necrosis factor-alpha".mp. or exp tumor necrosis factor/ (353070)

9 ("tumor necrosis factor-alpha" or "tumour necrosis factor-alpha").mp. (239192)

10 tumor necrosis factor alpha antibody.mp. or exp tumor necrosis factor antibody/

(11119)

11 ("tumo?r necrosis factor-alpha" or "tumo?r necrosis factor-α").ti,ab. (172992)

12 ("tumor necrosis factor-α" or "tumour necrosis factor-α").mp. (414343)

13 ("tumor necrosis factor alpha antibody" or "tumor necrosis factor-α

antibody").mp. (11170)

14 ("tumo?r necrosis factor alpha antibody" or "tumo?r necrosis factor-α

antibody").ti,ab. (312)

15 ("tumo?r necrosis factor* inhibitor*" or "tumo?r necrosis factor* antibod*").ti,ab.

(2785)

16 ("tumour necrosis factor alpha antibody" or "tumour necrosis factor-α

antibody").mp. (59)

17 ("tumor necrosis factor*" or "tumour necrosis factor*").mp. (414612)

18 ("tumor necrosis factor antibody" or "tumour necrosis factor antibody").mp. (5248)

19 "tumo?r necrosis factor?".ti,ab. (173537)

20 ("tumo?r necrosis factor alpha antibody" or "tumo?r necrosis factor-α

antibody").ti,ab. (312)

21 ("tumo?r necrosis factor* inhibitor*" or "tumo?r necrosis factor* antibod*").ti,ab.

(2785)

22 "anti-tumo?r necrosis factor*".ti,ab. (7812)

23 ("tumor necrosis factor antibody" or "tumour necrosis factor antibody").mp. (5248)

24 ("tumor necrosis factor* inhibitor*" or "tumour necrosis factor* inhibitor*").mp.

(15711)

25 "anti‐TNF‐alpha monoclonal antibod*".ti,ab. (1)

26 anti tnf*.ti,ab. (26866)

27 anti-tnf*.ti,ab. (26866)

28 antitnf*.ti,ab. (449)

29 etanercept.mp. or exp etanercept/ (32945)

30 (TNFR:Fc or etanercept or p75-Fc or Enbrel).ti,ab. (15175)

31 certolizumab pegol.mp. or exp certolizumab pegol/ (6959)

32 (certolizumab or CDP870 or Cimzia).ti,ab. (3450)

33 golimumab.mp. or exp golimumab/ (7407)

34 (golimumab or CNTO 148 or simponi).ti,ab. (3875)

35 infliximab.mp. or exp infliximab/ (52907)

36 (infliximab or cA2 or TA-650 or remicade).ti,ab. (108239)

37 adalimumab.mp. or exp adalimumab/ (35347)

38 (adalimumab or humira or d2e7).ti,ab. (15039)

39 8 or 9 or 10 or 11 or 12 or 13 or 14 or 15 or 16 or 17 or 18 or 19 or 20 or 21 or

22 or 23 or 24 or 25 or 26 or 27 or 28 or 29 or 30 or 31 or 32 or 33 or 34 or 35 or 36 or 37 or 38 (546895)

40 nuclear magnetic resonance imaging.mp. or exp nuclear magnetic resonance

imaging/ (997839)

41 (MR imag* or magnetic resonance imag* or MRI).ti,ab. (637864)

42 40 or 41 (1057984)

43 7 and 39 and 42 (1914)

44 limit 43 to yr="2000 -Current" (1911)

**Cochrane Library** (n=267):

1 MeSH descriptor: [Spondylarthropathies] explode all trees (1164)

2 MeSH descriptor: [Spondylitis, ankylosing] explode all trees (688)

3 MeSH descriptor: [Spondylitis] explode all trees (1376)

4 (ankylosing spondylitis):ti,ab,kw (2013)

5 (spondylitis):ti,ab,kw (2153)

6 (Spondylarthropathies):ti,ab,kw (78)

7 (ankylos$ or spondyl$):ti,ab,kw (2161)

8 (bekhterev$ or bechterew$):ti,ab,kw (16)

9 (Marie adj struempell$):ti,ab,kw (0)

10 #1 OR #2 OR #3 OR #4 OR #5 OR #6 OR #7 OR #8 OR #9 (2779)

11 MeSH descriptor: [tumor necrosis factor-alpha] explode all trees (3075)

12 (tumo?r necrosis factor-alpha):ti,ab,kw (6399)

13 (tumo?r necrosis factor-α):ti,ab,kw (1346)

14 (tumo?r necrosis factor alpha antibody):ti,ab,kw (952)

15 (tumo?r necrosis factor-α antibody):ti,ab,kw (103)

16 (tumo?r necrosis factor?):ti,ab,kw (11394)

17 (tumo?r necrosis factor antibod*): ti,ab,kw (1643)

18 (tumo?r necrosis factor* inhibitor*):ti,ab,kw (2575)

19 (anti-tumo?r necrosis factor*):ti,ab,kw (683)

20 (anti-TNF-alpha monoclonal antibod*):ti,ab,kw (83)

21 (anti tnf*):ti,ab,kw (4794)

22 (antitnf*):ti,ab,kw (2017)

23 MeSH descriptor: [Etanercept] explode all trees (760)

24 (etanercept or enbrel) :ti,ab,kw (2193)

25 (certolizumab or CDP870 or cimzia):ti,ab,kw (650)

26 (golimumab or "CNTO 148" or simponi):ti,ab,kw (674)

27 (infliximab or cA2 or TA-650 or remicade):ti,ab,kw (2888)

28 MeSH descriptor: [Infliximab] explode all trees (725)

29 (adalimumab or humira or d2e7):ti,ab,kw (3033)

30 MeSH descriptor: [Adalimumab] explode all trees (746)

31 #11 OR #12 OR #13 OR #14 OR #15 OR #16 OR #17 OR #18 OR #19 OR #20 OR #21 OR #22 OR #23 OR #24 OR #25 OR #26 OR #27 OR #28 OR #29 OR #30 (19105)

32 MeSH descriptor: [magnetic resonance imaging] explode all trees (7736)

33 (nuclear magnetic resonance imaging):ti,ab,kw (9940)

34 (magnetic resonance imaging):ti,ab,kw (24345)

35 (MR imag* or magnetic resonance imag* or MRI):ti,ab,kw (33656)

36 #32 OR #33 OR #34 OR #35 (33759)

37 #10 AND #31 AND 36 (267=reviews 2+trials 265)
