# Supplementary material for: Impact of tumor necrosis factor α inhibitors on MRI inflammation in axial spondyloarthritis assessed by Spondyloarthritis Research Consortium Canada score: A meta-analysis
Source: PLoS One. 2020 Dec 31;15(12):e0244788. doi: 10.1371/journal.pone.0244788 (PMC7775088; doi:10.1371/journal.pone.0244788)
Supplement: S1 Table — (DOCX) [file pone.0244788.s003.docx]

| **S1 Table. Sensitivity analysis: Pooled data of comparing TNFi versus placebo** | | | | | | | |
| --- | --- | --- | --- | --- | --- | --- | --- |
| **Outcomes**  **(TNFi vs. Placebo)** | **Study number** | **Total patient number** | | **Heterogeneity** | | **MD (95%CI)** | **P value** |
|  |  | **TNFi** | **Control** | **I^2^** | **P** |  |  |
| SPARCC of sacroiliac joint | 9 | 647 | 612 | 16% | 0.30 | 2.89[2.60, 3.18] | P<0.00001^*^ |
| SPARCC of spine | 4 | 251 | 262 | 87% | P＜0.0001 | 1.86[1.26,2.46] | P<0.00001^*^ |
| ASDAS | 5 | 329 | 338 | 87% | P<0.00001 | 0.97[0.70, 1.25] | P<0.00001^*^ |
| BASDAI | 6 | 488 | 496 | 77% | 0.0005 | 0.95[0.52, 1.38] | P<0.0001^*^ |
| BASFI | 5 | 465 | 473 | 62% | 0.03 | 0.89[0.55, 1.23] | P<0.00001^*^ |
| CRP | 5 | 465 | 473 | 65% | 0.02 | 2.89[0.33, 5.45] | P=0.03^*^ |
| Abbreviations: SPARCC, Spondyloarthritis Research Consortium Canada score; TNFi, tumor necrosis factor α inhibitor; ASDAS, Ankylosing Spondylitis Disease Activity Score; BASDAI, Bath Ankylosing Spondylitis disease activity index; BASFI, Bath Ankylosing Spondylitis functional index; CRP, C-reactive protein; MD, mean difference; CI, confidence interval; *p<0.05. | | | | | | | |
